# Determination of L-Theanine and Caffeine Contents in Tea Infusions with Different Fermentation Degrees and Brewing Conditions Using the Chromatographic Method

**DOI:** 10.3390/foods14132313

**Published:** 2025-06-30

**Authors:** Gamze Ayakdaş, Duygu Ağagündüz

**Affiliations:** 1 Department of Nutrition and Dietetics, Acıbadem University, Kerem Aydınlar Campus, Ataşehir, Istanbul 34755, Turkey; gamze.ayakdas@acibadem.edu.tr; 2 Department of Nutrition and Dietetics, Gazi University, Emek, Ankara 06490, Turkey

**Keywords:** L-theanine, caffeine, tea, brewing, HPLC

## Abstract

This study aimed to determine the caffeine and L-theanine contents in tea infusions prepared under varying fermentation degrees, brewing conditions (temperature and time), and serving methods (tea bag/loose-leaf). Infusions of six tea types (green, white, yellow, oolong, black, and Pu-erh) and various serving forms of green, white, and black tea were brewed at 80 °C and 100 °C for 2, 5, and 10 min. Contents were analyzed using reversed-phase high-performance liquid chromatography. Caffeine levels were highest in black and yellow tea (14 ± 1.0 mg/200 mL and 13.8 ± 0.2 mg/200 mL, respectively), both of which were significantly higher than the levels in green, white, and oolong tea (11 ± 2.1 mg/200 mL; 5.8 ± 0.7 mg/200 mL; and 4 ± 1.6 mg/200 mL, respectively; *p* < 0.001), whereas Pu-erh tea (13 ± 2.9 mg/200 mL) had caffeine levels comparable to that of black tea (*p* > 0.05). L-Theanine levels were highest in black and green tea (12.3 ± 2.8 mg/200 mL and 12.5 ± 2.5 mg/200 mL, respectively), and these levels were significantly higher than those in the other tea types (*p* < 0.001). Brewing temperature only affected caffeine levels n, whereas L-oolong tea (4.4 → 13.5 mg/200 mL, *p* < 0.05); theanine remained stable (*p* > 0.05). Longer brewing times significantly raised both components (e.g., yellow tea caffeine levels rose to 53 ± 16.9 mg/200 mL at 10 min; *p* < 0.05). In black tea, cup-sized bags yielded higher caffeine levels (39 ± 9.9 mg/200 mL) than loose-leaf (24 ± 7.2 mg/200 mL) and teapot bags (*p* < 0.001). Serving method had no effect on green and white teas (*p* > 0.05). In conclusion, fermentation rate, brewing conditions, and serving method were found to influence the caffeine and L-theanine levels of tea infusions.

## 1. Introduction

Tea, obtained from the fresh leaves of the *Camellia sinensis* (L.) *O. Kuntze* plant belonging to the Theaceae family, is the second most consumed beverage worldwide after water [1]. Depending on the processing techniques applied, six main types of tea are produced, including black, green, white, yellow, oolong, and Pu-erh [2,3]. These teas are classified according to the oxidation levels to which they are exposed during the production process: green and white tea undergo minimal oxidation, oolong and yellow tea undergo moderate oxidation, and black and Pu-erh tea undergo high oxidation [4]. The region where it is grown, climatic conditions, season, age and type of plant, and processing methods influence the chemical composition of tea [5]. Furthermore, consumer-related variables, including brewing container, water temperature, brewing time, and tea/water ratio, significantly affect the infusion composition [6].

Tea, derived from the *C. sinensis* plant, contains a wide range of phenolic compounds and volatile flavor constituents [1]. Current evidence has suggested that tea can exert protective effects against inflammatory, cardiovascular, gastrointestinal, diabetic, and neurodegenerative diseases, as well as some types of cancer and neurological disorders, including depression and cognitive decline [7]. L-theanine and caffeine are two prominent bioactive components that are associated with the formation of these effects [8].

Caffeine, a purine alkaloid with the structure of 1,3,7-trimethylxanthine, is naturally present in tea, coffee, and cocoa [9,10]. This slightly bitter-tasting compound is one of the main elements that provide the characteristic bitterness of tea and is recognized for its diuretic effect and energy metabolism-accelerating properties [11]. Its effects may vary according to age, sex, nutritional status, and amount of consumption [12]. Low doses can positively affect cognitive performance and attention; however, when taken in high amounts, it can lead to negative consequences, including anxiety, insomnia, and cardiovascular disorders [9,13].

L-theanine, a nonproteinogenic amino acid detected only in the tea plant, is the main compound that provides the umami flavor of green tea [14]. In addition to its role in balancing the bitterness caused by catechin and caffeine, it possesses diverse health benefits, including sedative effect, antitumor activity, cardiovascular disease prevention, and neuroprotective properties. It may positively contribute to cognitive performance by increasing attention span [14,15]. L-theanine works by inhibiting glutamate reuptake, antagonizing low-affinity glutamate receptors in the hippocampus, and providing neurological protection via gamma-aminobutyric acid (GABA)-A receptors [10].

The levels of these bioactive components in tea significantly vary, especially depending on the degree of fermentation. Metabolomic studies have revealed that L-theanine, caffeine, and catechin levels substantially vary in different tea types [11]. The L-theanine level is generally higher in unfermented teas (green tea), which tends to decrease as fermentation progresses [6]. Although the lack of steaming can increase the L-theanine level, trace amounts of this compound have also been reported in some green tea types. In contrast, high L-theanine levels were observed in some black tea samples, suggesting the presence of other factors affecting tea composition [4].

Caffeine, a more stable component among the tea types, is largely unaffected by fermentation [4]. However, environmental factors, including the region where it is grown, climate, and harvest time, may cause changes in the caffeine level [16]. Conversely, brewing parameters, including the brewing time, temperature, water ratio, degree of leaf breakdown, stirring, and milk addition, are also determinants of caffeine and L-theanine levels in infusions [6,17,18]. Generally, higher temperatures and longer brewing times increase the extraction of these components but may also cause degradation of some polyphenols [19,20,21]. Therefore, optimizing the brewing conditions plays a significant role in maximizing the health effects of tea [22,23].

Previous studies have determined the L-theanine and caffeine levels in various tea types, and some studies have also evaluated the effects of brewing conditions on these components [6,24,25,26,27]. It has been shown that L-theanine levels can vary depending on the tea source and processing steps [26]. In another study, these two compounds were investigated using the high-performance liquid chromatography (HPLC) method, and significant differences were observed between the samples [4]. However, studies systematically evaluating L-theanine and caffeine levels in tea infusions with different fermentation degrees in the Turkish market are scarce. Therefore, this study aimed to determine caffeine and L-theanine levels in tea infusions with different fermentation degrees under different brewing conditions using the HPLC method. The findings may contribute to preclinical and clinical research on the bioactive component profile of tea.

## 2. Materials and Methods

### 2.1. Sample Selection and Procurement

In this study, six different tea types with different fermentation degrees obtained from physical markets and online sales platforms in Istanbul between July and September 2023 were evaluated. These were categorized as green tea (nonfermented), white tea (nonfermented), yellow tea (partially fermented), oolong tea (partially fermented), black tea (fermented), and Pu-erh tea (intensely fermented). Samples were obtained ready-to-consume in bags or loosely packaged in their original packages. For black tea alone, this study also included tea bag tea samples. However, for the other tea types, the tea bag tea form was not available on the market and could not be examined.

Overall, 68 tea samples were evaluated for caffeine analysis. For L-theanine analysis, 20 tea samples selected from these samples were used. To ensure homogenization in all samples, label information was investigated, and samples harvested in the same season and with a difference of ±1 month between production dates were preferred. A detailed list of samples is presented in Table 1. As shown in Table 1, each of the 68 samples was obtained from a different commercial brand and represents an independent tea product.

### 2.2. Preparation of Tea Infusions

For caffeine and L-theanine analyses, infusions were prepared by applying the experimental brewing procedure. Ultrapure water was obtained using the New Human Power I Scholar UV device. For loose-leaf samples, 2 g each of dry tea were brewed with 200 mL of water in a sealed glass herbal tea brewing container. Tea bags (2 g) were dipped 5–6 times in 200 mL of water and subsequently brewed again in a sealed container.

Each sample was separately brewed at two different temperatures (80 °C and 100 °C) and three different times (2, 5, and 10 min) [21,22,28,29,30]. Two infusions were prepared for each brewing condition, and these duplicate measurements were averaged.

**Figure 1:**
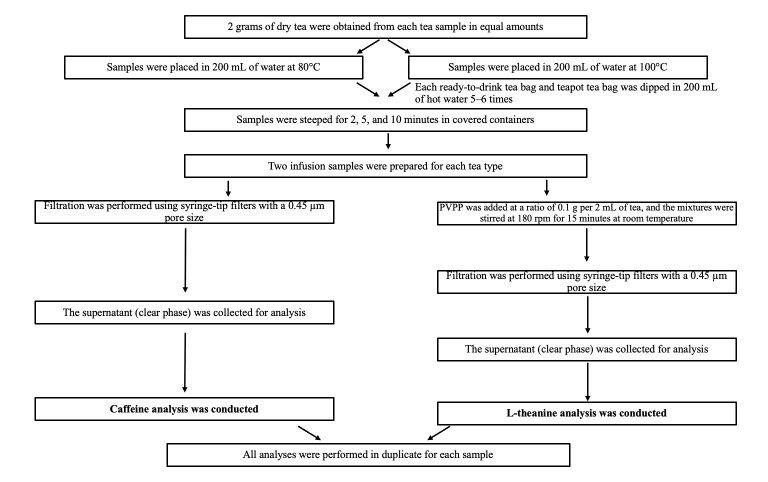
Experimental brewing procedure and analysis of the caffeine and L-theanine samples.

The temperatures were controlled using a Rossman™ kettle(Rossmann GmbH, Burgwedel, Germany)and an Arcone TP101™ thermometer (ARCO Instrument Co., Ltd., Shenzhen, China) The experimental brewing procedure and analysis of the samples are shown in Figure 1.

### 2.3. Chromatographic Analyses

The levels of caffeine and L-theanine in tea infusions were analyzed using reversed-phase high-performance liquid chromatography (RP-HPLC). For this analysis, we used a system consisting of a SpectraSYSTEM P1000 pump, a SpectraSYSTEM AS3000 autosampler, and a Thermo Finnigan UV1000 UV detector (Thermo Electron Corporation, San Jose, CA, USA), equipped with an SVEA C18 Core Shell column (2.6 µm, 150 × 3.0 mm; SVEA Analytical, Uppsala, Sweden).

#### 2.3.1. Caffeine

Caffeine analysis in tea infusions was filtered through 0.45 µm filters (Welch MCE [mixed cellulose ester] Syringe Filter, diameter: 25 mm) and transferred to 1.5 mL HPLC vials. Analyses were performed on an SVEA C18 Core Shell 2.6 µm 150 × 3.0 mm column with a water–methanol (60:40) mobile phase, 275 nm detection, 1 mL/min flow rate, and 40 °C column temperature. The injection volume was set at 100 µL, and the total run time was 5 min. The method was adapted from Shrestha et al. (2016) [31]. Duplicate analysis was performed for each sample.

#### 2.3.2. L-Theanine

L-theanine analysis in tea infusions was performed by adding polyvinylpolypyrrolidone (PVPP) to each sample at a ratio of 1 g PVPP per 20 mL of tea to remove interfering polyphenols. The mixtures were stirred using a VELP Scientifica ZX3 vortex mixer at 180 rpm for 15 min at room temperature. After treatment, the samples were filtered using 0.45 µm filters (Welch MCE (mixed cellulose ester) Syringe Filter, diameter: 25 mm), and the clear supernatants were transferred into 1.5 mL HPLC vials for injection. Analyses were performed on an SVEA C18 Core Shell 2.6µm 150 × 3.0 mm column with a water–methanol (75:25) mobile phase, 210 nm detection, 1 mL/min flow rate, and 25 °C column temperature for the analysis. The injection volume was set at 100 µL, and the total run time was 13 min. The method was adapted from Henríquez-Aedo et al. (2013) [32]. Duplicate analysis was performed for each sample.

#### 2.3.3. Method Validation

**Figure 2:**
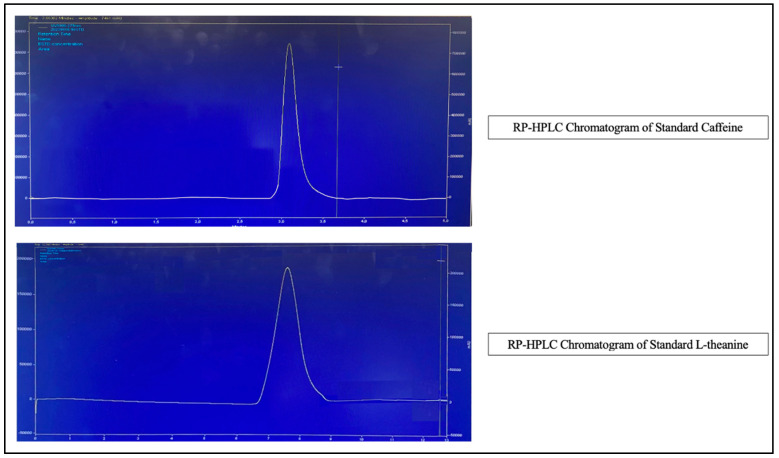
Representative reversed-phase high-performance liquid chromatography chromatograms of the standard caffeine and L-theanine solutions.

Caffeine standards (10–300 ppm) were prepared and diluted 1/10 prior to analysis. Similarly, L-theanine standards (25–125 ppm) were prepared and diluted according to the method requirements. The analysis was performed using a validated RP-HPLC method.The retention times and peak characteristics for both analytes are shown in Figure 2.

Linearity (R^2^) and coefficient of variation (CV%) were calculated as part of the validation of the HPLC methods used for caffeine and L-theanine level determination. R^2^ = 0.9995 for caffeine and R^2^ = 0.9999 for L-theanine, confirming the high reliability of the methods. Details are presented in the Supplementary Materials.

The repeatability of the method was evaluated by analyzing two independently prepared samples for each brewing condition, each measured in duplicates. The relative standard deviation (RSD) was calculated as 12.73% for caffeine and 2.82% for L-theanine, both of which fall within acceptable ranges for natural product analysis. Selectivity was ensured by confirming the retention times and peak shapes of caffeine and L-theanine in real tea samples compared with standard solutions under the same chromatographic conditions, without interference from matrix components.

The limit of detection (LOD) and limit of quantification (LOQ) were calculated based on the residual standard deviation of the response and the slope of the calibration curve. The calculated LOD and LOQ values were 0.13 mg/L and 0.40 mg/L for caffeine and 0.009 mg/L and 0.027 mg/L for L-theanine, respectively. These low detection thresholds confirm the sensitivity of the method.

Recovery (accuracy) tests could not be performed due to the lack of certified blank matrices and the unavailability of suitable reference materials. Since caffeine and L-theanine are naturally present in all real tea samples, it was not feasible to conduct spike-recovery tests under current laboratory conditions. Likewise, the robustness evaluation could not be completed due to limited access to multiple instruments, operators, or deliberate variation studies.

### 2.4. Statistical Analysis

Data were analyzed using IBM SPSS v25.0 and Jamovi v2.3, with the WRS2 package employed for robust statistical procedures in Jamovi. Descriptive statistics were reported as the arithmetic mean ± the standard deviation ($\bar{x}$ ± SD) or the arithmetic mean ± the standard error ($\bar{x}$ ± SE), depending on the objective. The SE was preferred particularly in cases where comparisons of means were the primary focus, in accordance with statistical consultation [33]. The normality of the distribution for caffeine and L-theanine levels was assessed using the Shapiro–Wilk test. For non-normally distributed data, a robust analysis of variance (ANOVA) was applied, whereas normally distributed data were analyzed using a three-way ANOVA. To compare caffeine and L-theanine levels between tea types with varying fermentation degrees but brewed under the same conditions (temperature and time), one-way ANOVAs and independent samples t-tests were used. The Bonferroni correction was applied for multiple comparisons. Additionally, to evaluate potential interaction effects between brewing temperature and time, a three-way ANOVA including interaction terms among fermentation degree, brewing temperature, and brewing time was conducted. Where significant interactions were observed, simple main effects were further examined. Significance levels of α = 0.05 and α = 0.001 were used throughout, and *p*-values equal to or below these thresholds were interpreted as statistically significant. The α = 0.05 level was used as the primary threshold for statistical significance; α = 0.001 was only applied in selected comparisons to denote highly significant results.

## 3. Results

In this study, caffeine and L-theanine levels in tea infusions with different fermentation degrees and brewing conditions were systematically evaluated. The findings are presented under subheadings according to variables, including the tea type, brewing temperature, brewing times, and serving methods.

The results are presented in four sequential subsections according to the study variables: tea type, brewing temperature, brewing time, and serving method. Although the effects of individual variables are discussed in each subsection, it is important to note that the potential interactions between fermentation degree, brewing temperature, and time were also evaluated using a three-way ANOVA. Therefore, results reflecting the combined effects of two or more parameters (e.g., temperature × time) are clearly indicated. This structure has been adopted to prevent confusion and ensure that the results are interpreted transparently.

### 3.1. Caffeine

#### 3.1.1. Caffeine Levels by Tea Type

**Figure 3:**
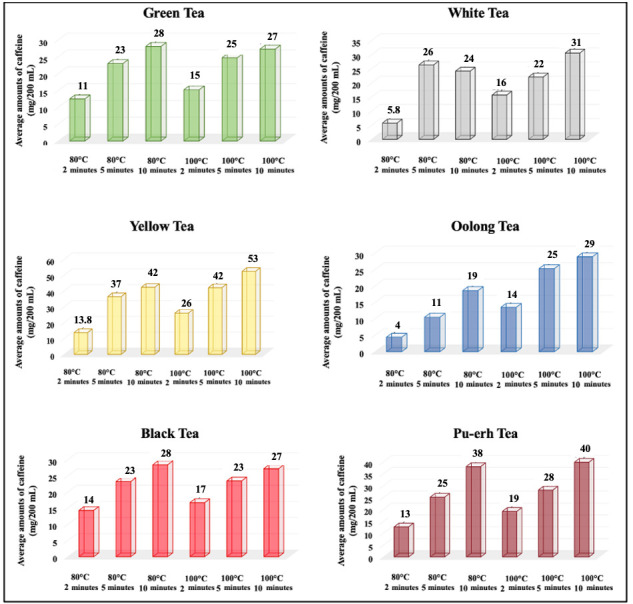
Comparison of caffeine levels in tea infusions brewed under different conditions with the same fermentation rates.

The mean ($\bar{x}$), standard error (SE), and CV values (%) of caffeine levels (mg/200 mL) in tea infusions brewed under the same conditions with different fermentation degrees are presented in Table 2. Moreover, the average caffeine levels across the tea types according to the brewing conditions are compared in Figure 3.

No statistically significant differences were observed among yellow tea (13.8 ± 0.2 mg/200 mL), black tea (14.0 ± 1.0 mg/200 mL), and Pu-erh tea (13.0 ± 2.9 mg/200 mL) brewed at 80 °C (*p* > 0.05); however, these three tea types exhibited significantly higher caffeine levels compared to green (11.0 ± 2.1 mg/200 mL), white (5.8 ± 0.7 mg/200 mL), and oolong (4.0 ± 1.6 mg/200 mL) teas (*p* < 0.001). When the brewing times were increased to 5 and 10 min, caffeine levels significantly increased across all the tea types (*p* < 0.05). The highest caffeine level was observed in yellow tea (53 ± 16.9 mg/200 mL) when brewed at 100 °C for 10 min (*p* > 0.001).

#### 3.1.2. Effects of Brewing Conditions on Caffeine Levels

Brewing temperature made a statistically significant difference only in oolong tea (*p* < 0.05). At 2 min of brewing, the caffeine level increased from 4 ± 1.6 mg/200 mL at 80 °C to 14 ± 3.9 mg/200 mL at 100 °C. Although temperature-dependent differences were observed in other tea types, these changes were not statistically significant (*p* > 0.05).

In green, white, yellow, and oolong teas, the caffeine levels obtained at a 2 min brewing time were significantly lower (*p* < 0.05) than those acquired at 5 and 10 min of brewing. However, no significant difference was observed across the values between 5 and 10 min (*p* > 0.05). In black and Pu-erh teas, significant increases in the caffeine levels were noted as the brewing time increased (*p* < 0.05). The comparison of the average caffeine levels in tea infusions brewed under different brewing conditions with the same fermentation rates is depicted in Figure 3.

#### 3.1.3. Effect of the Serving Method on Caffeine Levels

The mean ($\bar{x}$) and standard deviation (SD) values of the caffeine level (mg/200 mL) in tea infusions under the same brewing conditions (brewing temperature and time) according to the serving method (tea bag, teapot bag tea [for black tea only], or loose-leaf) are shown in Table 3.

In green teas, following 5 min of brewing at 80 °C, the tea bags contained 28 ± 5.6 mg/200 mL of caffeine, whereas the loose-leaf contained 17 ± 8.9 mg/200 mL. The difference was statistically significant (*p* < 0.05). In black teas, following 10 min of brewing at 100 °C, the tea bags had 39 ± 9.9 mg/200 mL of caffeine, whereas the loose form had 24 ± 7.2 mg/200 mL. This difference was statistically significant (*p* < 0.05). In white teas, different serving methods did not show significant differences in terms of caffeine levels (*p* > 0.05).

### 3.2. L-Theanine

#### 3.2.1. Caffeine Levels by Tea Type

**Figure 4:**
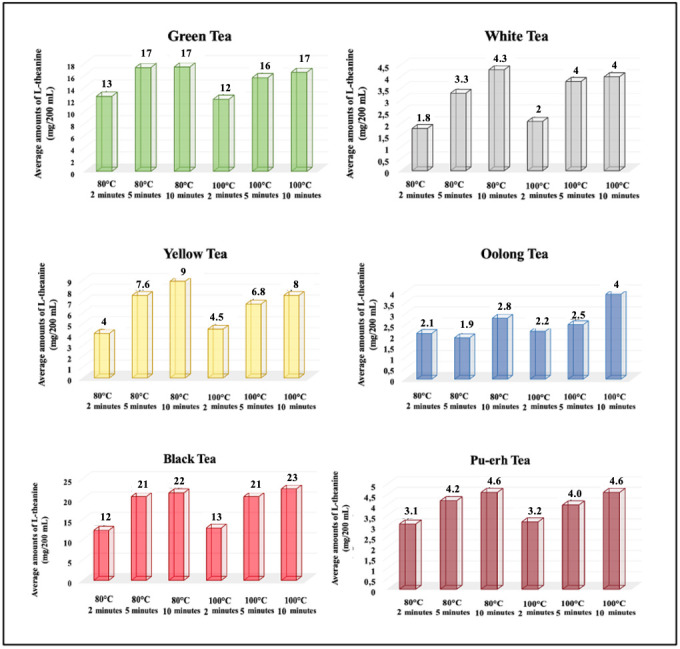
Comparison of the mean L-theanine levels contained in tea infusions brewed under different brewing conditions with the same fermentation rates.

The mean ($\bar{x}$), SE, and CV values (%) of L-theanine levels (mg/200 mL) in tea infusions brewed under the same conditions with different fermentation rates are presented in Table 4. Moreover, the mean L-theanine levels in tea infusions brewed under the same brewing conditions are compared in Figure 4.

Black tea (12 ± 2.8 mg/200 mL) and green tea (13 ± 2.5 mg/200 mL) showed the highest L-theanine levels in infusions brewed at 80 °C for 2 min (*p* > 0.001). These teas contained significantly more L-theanine than white (1.8 ± 0.4 mg), oolong (1.8 ± 0.5 mg), yellow (4 ± 1.1 mg), and Pu-erh (3.1 ± 0.4 mg) teas (*p* < 0.001).

#### 3.2.2. Effects of Brewing Conditions on L-Theanine Levels

The brewing temperature-induced difference was not statistically significant (*p* > 0.05). As the brewing time increased, L-theanine levels significantly increased in green, white, yellow, and black teas; however, no significant difference was observed between 5 and 10 min (*p* > 0.05). Oolong and Pu-erh teas showed significant increases between 2 and 10 min (*p* < 0.05). The comparison of the average L-theanine levels in tea infusions brewed under different brewing conditions with the same fermentation rates is shown in Figure 4.

#### 3.2.3. Effects of the Serving Method on L-Theanine Levels

The mean ($\bar{x}$) and SD values of L-theanine levels (mg/200 mL) under the same brewing conditions (brewing temperature and time) according to the serving method (bag tea or loose-leaf) are provided in Table 5.

In green teas, following 10 min of brewing at 100 °C, glass tea bag infusions contained an average of 20.5 ± 0.1 mg/200 mL of L-theanine, whereas loose-leaf contained 13 ± 4.8 mg/200 mL. However, the difference was not statistically significant (*p* > 0.05). Similarly, in black teas, although differences were observed between loose and bagged teas in all brewing conditions, these differences were not statistically significant (*p* > 0.05).

## 4. Discussion

Tea from the leaves of the *C. sinensis* plant is divided into six main types according to the processing steps; withering, curling, fermentation, stabilization, and drying steps determine the chemical profile of the tea [1,34]. Although caffeine is generally considered chemically stable during tea fermentation, microbial fermentation and enzymatic oxidation may affect its extractability, leading to higher caffeine levels in highly fermented teas such as Pu-erh and black tea—especially when evaluated on a dry-weight basis. Conversely, L-theanine levels may decrease due to oxidative degradation [35,36,37]. However, low L-theanine levels in some green teas and high levels in some black teas suggest that environmental and agricultural factors other than processing are also influential [6,38]. The prevalence of hot infusion as a serving method and variables, including brewing time, temperature, and consumption form, can affect the bioactive content of tea [17,39]. In this context, this study comprehensively evaluated the effects of different tea types and brewing variables on caffeine and L-theanine levels.

In the literature, the number of studies simultaneously investigating six different tea types is highly limited [40,41]. Most studies have focused on only one or a few tea species [42,43,44,45,46]. Similar to the present study, a study involving six tea types demonstrated a slight decrease in caffeine content as the fermentation rate increased in teas produced from leaves harvested at the same harvest; however, overall, the caffeine levels of all teas were similar. In contrast, L-theanine levels were the highest in unprocessed tea leaves and decreased with increasing fermentation. Notably, Pu-erh tea exhibited significantly lower L-theanine levels [40]. In another study, when fresh leaves and six different tea types were compared, the lowest caffeine levels were observed in fresh leaves (29.80 ± 0.25 mg/g) and the highest in white (33.43 ± 0.42 mg/g) and Pu-erh teas (33.71 ± 0.53 mg/g). Regarding L-theanine, fresh leaves showed the highest concentration (1.00 mg/g), whereas Pu-erh (0.30 mg/g) and white tea (0.63 mg/g) had the lowest, respectively [41].

In the literature, caffeine levels are relatively consistent across tea types, whereas L-theanine levels are highly variable. For example, in studies by Boros et al. (2016) and Horrani et al. (2013), L-theanine levels were extremely low in Pu-erh teas, whereas a significant variation was noted in green teas [4,47]. This inconsistency in L-theanine levels has been attributed to several factors, including the region where the tea is grown, processing method, harvest time, storage conditions, and analytical approaches. Furthermore, parameters, including the brewing temperature, duration, tea–water ratio, and mode of consumption, significantly influence the tea composition [6,17,47].

In this study, a caffeine content of 11.4–27.3 mg/200 mL and an L-theanine content of 12.0–17.4 mg/200 mL were detected in the green tea infusions. Similarly, in the literature, the caffeine content of green teas is reported in the range of 11.0–20.0 mg/g; Indian- and Chinese-origin teas contain higher caffeine levels than Japanese-origin teas [44]. Yi et al. (2015) identified green tea as the species with the highest caffeine content [48]. Regarding L-theanine, some sources have indicated that green and white teas have higher levels, whereas Keenan et al. (2011) emphasized black teas [6,24,27,49]. The findings of this study are consistent with some of these views, with the highest L-theanine levels noted in black and green teas. The variations in the results are believed to be due to the geographical origin of the teas, climatic conditions, harvest time, and processing methods.

In this study, the white tea infusions had caffeine and L-theanine levels of 5.8–30.6 and 1.8–4.3 mg/200 mL, respectively. Some studies have reported that white teas contain higher caffeine levels than green teas [43,49], whereas green teas generally have higher L-theanine levels [50]. The findings of this study also support this trend regarding L-theanine. The low L-theanine level in white teas is attributed to oxidative reactions caused by Polyphenol oxidase (PPO) and peroxidase (POD) enzymes that remain active during the withering stage; therefore, white teas are partially similar to fermented teas in terms of processing [40,51].

In this study, the yellow tea infusions had caffeine and L-theanine levels of 13.8–52.5 and 4.1–8.9 mg/200 mL, respectively. Although studies on yellow tea are limited, some studies have suggested that yellow teas can contain similar or higher caffeine levels than green and black teas [51]. Regarding L-theanine, yellow teas are generally reported to contain lower levels than green teas [52]. The “yellowing” process employed in yellow tea production can lead to a reduction in both components [53], and this trend was confirmed in this study in terms of L-theanine; however, high caffeine levels were noted. It is believed that these differences may be due to factors, including the region where the tea is grown, processing conditions, and genetic variations.

In this study, the caffeine and L-theanine levels in the oolong tea infusions were 4.4–28.9 and 1.9–3.9 mg/200 mL, respectively. In the literature, oolong teas are characterized by varying caffeine and L-theanine levels, resulting from their fermentation rates ranging from 10% to 80% [4,47,54]. High caffeine levels have been reported in some studies and very low L-theanine levels in some samples [42]. Consistent with the literature, oolong teas exhibited the lowest caffeine levels in this study; it is believed that this difference may be due to the fact that the products used in the sample do not adequately reflect the fermentation rates and the limited variety of teas available in the Turkish market [55].

In this study, the black tea infusions had caffeine and L-theanine levels of 14.3–27.2 and 12.3–22.5 mg/200 mL, respectively. In the literature, black teas generally contain the highest caffeine levels (25.7–34.2 mg/g), mostly with samples obtained from production regions, such as China, India, Japan, and Sri Lanka [42,44,45,56,57]. However, most of the black tea samples analyzed in this study were from Turkey and had shorter brewing times (2–10 min), which may have caused the lower values [46,58]. The L-theanine levels obtained support the high content of Turkish black teas and align with the high values of 24.2 ± 5.7 mg/200 mL reported by Keenan et al. Although green teas contain higher L-theanine levels in some sources, black teas emerged with higher levels in this study and were consistent with the findings in the literature [6,54,59].

In this study, the Pu-erh tea infusions had caffeine and L-theanine levels of 12.7–39.9 and 3.1–4.6 mg/200 mL, respectively. In the literature, the findings on the caffeine content of Pu-erh teas are contradictory; some studies have indicated that the long fermentation process does not affect the caffeine level, whereas others have reported that the caffeine level decreases during this process [60,61]. Conversely, regarding L-theanine, most studies have demonstrated that prolonged fermentation significantly reduces the content of this component [27,37]. In the present study, low L-theanine levels were observed, which is consistent with previous studies in which no L-theanine or very low levels of 0.07–0.26 mg/g were noted in Pu-erh tea [4,47]. These differences are believed to be due to variations in production methods in addition to the advanced fermentation of Pu-erh teas.

In this study, a general tendency for increased caffeine levels with increasing brewing temperature was observed in all tea types; however, this difference was statistically significant only in oolong teas. Similarly, it has been reported in the literature that caffeine levels increased in parallel with the temperature in infusions prepared at 60 °C, 80 °C, and 100 °C for 5 min [44]. In the study by Pan et al. (2018), the caffeine content increased as the temperature increased in white tea infusions, whereas L-theanine levels moderately increased in some samples and decreased in others in the 90–100 °C range [62]. Keenan et al. (2011) reported that the brewing temperature did not significantly affect the L-theanine content, with the main dissolution occurring within the first 5 min [6]. These results may be attributed to the temperature-sensitive solubility of caffeine, which increases its migration to the brew, whereas L-theanine maintains its stable structure at 60–100 °C. Moreover, parameters, including the width of the temperature range, brewing time, and tea–water ratio, affect the amount of bioactive components in the tea. The fact that the infusion brewing temperatures and times used in this study reflect actual consumption habits strengthens the association of the results obtained with the consumption patterns in practice and indicates that the findings are generally consistent with the literature. Furthermore, it should be noted that single-infusion brewing procedures, as applied in our study, may not ensure complete extraction of caffeine from tea leaves. Previous studies have demonstrated that multiple extractions with hot water (≥90 °C) are necessary to fully extract caffeine and related xanthines [44,63]. Therefore, the caffeine levels reported here represent those typically obtained under household brewing practices and do not reflect the total caffeine content present in the dry tea material.

In preparing tea infusions, tea types and brewing times varying according to cultural, and individual preferences can be applied [64]. Brewing time affects the amount of caffeine in the tea infusions [57]. In this study, the effect of brewing time on the passage of caffeine and L-theanine varied depending on the tea type; the general trend was an increase in the passage of both components into the infusion with increasing brewing time. However, this increase tended to stabilize after a certain point. Significant differences were observed between 2 min and 5 and 10 min brewing times, especially for green, white, and yellow teas, whereas for oolong, black, and Pu-erh teas, this difference was more pronounced for both caffeine and L-theanine. These findings suggest that brewing time is a crucial parameter but should be considered along with the type and temperature. Similarly, in the literature, caffeine concentration increased with brewing time in high-temperature constant water brews; however, this increase slowed down past 3–5 min [64,65]. For example, Turkish green teas reach the highest caffeine level after 3 min of brewing at 85 °C, with no significant difference at longer times [65]. The L-theanine transition was noted to be sensitive to the brewing time; the increase was generally intense in the first minutes but showed less sensitivity to temperature change [47]. In Turkish culture, where black teas are traditionally brewed for 15–25 min, the brewing times of 2, 5, and 10 min applied in this study are shorter than the actual consumption conditions, which explains the relatively low findings compared with the high values in the literature [58].

The serving method represents a crucial variable that can affect the chemical composition of tea infusions; however, studies on this topic are very limited. In the present study, among cup-bag, tea bag, and bulk forms, only black teas exhibited statistically significant differences in caffeine levels, with cup-bag teas containing the highest caffeine levels. Conversely, no significant effect of serving type on both caffeine and L-theanine contents was noted in white and green teas. There are results in the literature that support these findings. Although some studies have reported variability in caffeine levels between bagged and loose green teas [17]; in most cases, these differences were not statistically significant [57]. Regarding L-theanine, no difference was observed between tea bags and bulk teas [6]. Differences in component levels between tea consumption forms may arise from both technical and user-related factors, including leaf particle size, bag material and pore structure, sachet size, and consumer habits (e.g., squeezing or repeated immersion). The literature has demonstrated that although small particles facilitate faster dissolution, larger bags may enhance compound diffusion [64]. In this study, the observed differences between cup and teapot bags may be attributed to leaf cut and bag material variations, as smaller particles in tea bags can accelerate infusion and result in component levels comparable to or exceeding those of loose-leaf.

## 5. Conclusions

This study revealed that the caffeine and L-theanine levels contained in tea infusions vary depending on several variables, including the tea type (fermentation rate), brewing conditions (temperature and time), and serving methods. Regarding caffeine levels, yellow, black, and Pu-erh teas were the tea types with the highest values, and these types contained statistically significantly more caffeine than green, white, and oolong teas. Regarding L-theanine levels, black and green teas were the most prominent; no significant difference was observed between white, oolong, and Pu-erh teas. Brewing temperature emerged as a determining factor on caffeine passage, and samples brewed at 80 °C and 100 °C, especially in oolong teas, demonstrated significant differences. However, L-theanine levels remained stable despite temperature changes. Brewing time led to increased caffeine and L-theanine levels. However, in some samples, particularly in white teas, this increase stabilized in certain periods. The serving method was identified as another crucial factor influencing caffeine levels, with tea bag infusions demonstrating higher caffeine levels. In contrast, L-theanine levels remained similar regardless of the serving method. These results suggest that the bioactive component profile of tea is influenced by several factors and that proper management of these factors can result in healthier and more functional tea consumption. Of note, parameters, including the tea type, brewing time, and temperature, should be considered, particularly for consumers who desire to optimize the amounts of health-related compounds, including caffeine and L-theanine.

## 6. Limitations

This study had some limitations. First, the availability of tea types with different fermentation degrees in the Turkish market prevented a homogeneous distribution in the sample selection. This has been a limitation in conducting comparative analyses across the tea types. Second, the diversity of methods used in the literature for analyzing tea constituents (different chromatographic techniques and extraction protocols) and sampling differences (dry leaf vs. infusion) make direct comparison of results challenging. Therefore, the findings of this study may partially contradict some results in the literature. Finally, the large number of parameters analyzed and the inability to control for exogenous variables (climatic conditions and processing techniques) increased the potential for variability. Future studies should clearly specify variables, including standardized brewing protocols, tea–water ratios, harvest conditions, and the region of cultivation, to ensure data comparability and a more accurate evaluation of the health effects of tea. Furthermore, the effects of tea infusions on human health should be investigated clinically.

## Figures and Tables

**Table 1:** Tea samples provided for caffeine and L-theanine level determination.

Caffeine		L-theanine	
Tea Types	Brand	Tea Types	Brand
**Green Tea (n:12)**		**Green Tea (n:4)**	
Loose-leaf (n:6)	A (n:1), C (n:2), D (n:1), E (n:1), K (n:1)	Loose-leaf (n:2)	D (n:1), E (n:1)
Cup of Tea Bags (n:6)	A (n:2), B (n:1), C (n:1), D (n:1), E (n:1)	Cup of Tea Bags (n:2)	D (n:1), E (n:1)
**White Tea (n:5)**		**White Tea (n:3)**	
Loose-leaf (n:3)	E (n:1), F (n:1), G (n:1)	Loose-leaf (n:2)	E (n:1), F (n:1)
Cup of Tea Bags (n:2)	A (n:1), L (n:1)	Cup of Tea Bags (n:1)	A (n:1)
**Yellow Tea (n:2)**		**Yellow Tea (n:2)**	
Loose-leaf (n:2)	F (n:1), L (n:1)	Loose-leaf (n:2)	F (n:1), L (n:1)
**Oolong Tea (n:4)**		**Oolong Tea (n:3)**	
Loose-leaf (n:3)	F (n:1), G (n:1), L (n:1)	Loose-leaf (n:2)	F (n:1), G (n:1)
Cup of Tea Bags (n:1)	F (n:1)	Cup of Tea Bags (n:1)	F (n:1)
**Black Tea (n:41)**		**Black Tea (n:6)**	
Loose-leaf (n:19)	A (n:1), B (n:4), C (n:4), D (n:2), E (n:3), F (n:1), G (n:2), H (n:1), I (n:1)	Loose-leaf (n:3)	C (n:1), D (n:1), E (n:1)
Teapot Bag (n:10)	A (n:2), B (n:3), C (n:1), D (n:2), J (n:2)	Cup of Tea Bags (n:3)	C (n:1), D (n:1), E (n:1)
Cup of Tea Bags (n:12)	A (n:2), B (n:2), C (n:2), D (n:1), E (n:2), F (n:1), J (n:2)		
**Pu-erh Tea (n:4)**		**Pu-erh Tea (n:2)**	
Loose-leaf (n:4)	F (n:2), G (n:1), L (n:1)	Loose-leaf (n:2)	F (n:1), L (n:1)

Each brand code (A–L) corresponds to a unique commercial tea product purchased separately.

**Table 2:** Mean ($\bar{x}$), standard error (SE), and coefficient of variation (CV) values (%) for the comparison of caffeine levels (mg/200 mL) in tea infusions with the same fermentation rates brewed under different brewing conditions according to the fermentation rates of the teas *.

Tea Types	Brewing Temperature (°C)	Brewing Time (min)					
		2		5		10	
		$\bar{x}$ ± S_e_	CV (%)	$\bar{x}$ ± S_e_	CV (%)	$\bar{x}$ ± S_e_	CV (%)
Green Tea (n:12)	80	11 ± 2.1	63.8	23 ± 2.8	42.2	28 ± 4.6	56.7
	100	15 ± 2.5	57.0	25 ± 3.1	43.5	27 ± 4.4	55.8
White Tea (n:5)	80	5.8 ± 0.7	47.7	26 ± 10.0	78.2	24 ± 3.1	54.5
	100	16 ± 2.2	31.3	22 ± 4.1	31.2	31 ± 2.5	27.5
Yellow Tea (n:2)	80	13.8 ± 0.2	2.0	37 ± 3.3	12.8	42 ± 10.9	36.4
	100	26 ± 1.4	7.6	42 ± 3.7	12.4	53 ± 16.9	45.5
Oolong Tea (n:4)	80	4 ± 1.6	45.7	11 ± 2.1	40.0	19 ± 3.7	39.8
	100	14 ± 3.9	57.8	25 ± 4.9	38.7	29 ± 2.4	16.6
Black Tea (n:41)	80	14 ± 1.0	44.8	23 ± 1.4	38.6	28 ± 1.5	33.8
	100	17 ± 1.0	38.3	23 ± 1.3	35.6	27 ± 1.5	35.3
Pu-erh Tea (n:2)	80	13 ± 2.9	45.7	25 ± 5.1	40.5	38 ± 9.9	52.0
	100	19± 9.5	57.0	28 ± 8.6	43.5	40 ± 9.9	55.8

Robust ANOVA. Caffeine levels of tea infusions brewed under the same conditions with different fermentation rates *p* > 0.05. Caffeine levels of tea infusions brewed under different conditions with the same fermentation rates *p* < 0.05. * Owing to the large number of variables, statistical significance levels are not shown in the table and are provided in detail in the explanations of the table.

**Table 3:** Mean ($\bar{x}$), standard deviation (SD), and coefficient of variation (CV) values (%) of the mean caffeine levels (mg/200 mL) in tea infusions with different serving methods under the same brewing conditions.

Tea Type	Brewing Temperature (°C)	Brewing Time (min)	Cup Bag		Teapot Bag		Loose-Leaf		Test Statistics *	*p*
			$\bar{x}$ ± ss	CV (%)	$\bar{x}$ ± -ss	CV (%)	$\bar{x}$ ± ss	CV (%)		
Green Tea (n_CB_:6, n_LT_:6)	80	2	13 ± 5.00	38.1	-		10 ± 5.70	54.8	0.869	0.405
		5	28 ± 5.60	20.5	-		17 ± 8.90	52.7	2.505	**0.031**
		10	36 ± 7.90	22.0	-		19 ± 8.40	43.8	3.535	**0.005**
	100	2	15 ± 5.70	37.9	-		15 ± 7.40	47.9	−0.108	0.916
		5	28 ± 6.90	24.9	-		20 ± 9.90	49.9	1.591	0.143
		10	33 ± 10.90	33.5	-		22 ± 9.90	44.7	1.711	0.118
White Tea (n_CB_:2, n_LT_:3)	80	2	8 ± 2.00	25.2	-		4 ± 2.20	51.2	1.911	0.152
		5	18.2 ± 0.10	0.8	-		42 ± 30.70	73.8	−1.022	0.382
		10	22 ± 0.60	2.9	-		35 ± 20.40	58.6	−0.865	0.451
	100	2	15 ± 3.80	25.9	-		15 ± 6.10	39.8	−0.109	0.920
		5	18 ± 5.10	28.9	-		25 ± 7.10	28.1	−1.244	0.302
		10	28 ± 3.70	13.3	-		37 ± 10.40	28.4	−1.134	0.339
Black Tea (n_CB_:12, n_TPB_:10, n_LT_:19)	80	2	20 ± 5.00 ^a^	24.7	8 ± 2.50 ^b^	30.7	15 ± 4.10 ^c^	27.7	24.366	**<0.001**
		5	30 ± 4.50 ^a^	14.9	18 ± 3.40 ^b^	18.8	23 ± 6.40 ^c^	28.0	15.374	**<0.001**
		10	38 ± 8.00 ^a^	21.2	25 ± 4.70 ^b^	18.7	26 ± 7.10 ^b^	27.5	12.930	**<0.001**
	100	2	23 ± 6.10 ^a^	26.6	11 ± 2.10 ^b^	19.9	18 ± 4.70 ^c^	26.7	19.060	**<0.001**
		5	33 ± 7.10 ^a^	21.6	20 ± 2.10 ^b^	10.6	22 ± 5.80 ^b^	26.4	18.406	**<0.001**
		10	39 ± 9.90 ^a^	25.3	25 ± 3.50 ^b^	13.8	24 ± 7.20 ^b^	29.6	16.768	**<0.001**

* Independent samples *t*-test; $\bar{x}$ ± ss. One-way analysis of variance; $\bar{x}$ ± ss; ^a–c^ no difference is noted between the mean caffeine levels of the serving methods with the same letter. Teapot bags of green and white teas are not available on the market and therefore cannot be investigated. CB, cup of tea bags; TPB, teapot bag; LT, loose-leaf.

**Table 4:** Mean ($\bar{x}$), standard error (SE), and coefficient of variation (CV) values (%) for the comparison of L-theanine levels (mg/200 mL) in tea infusions with the same fermentation rates brewed under different brewing conditions according to the fermentation (oxidation) rates of the teas *.

Tea Types	Brewing Temperature (°C)	Brewing Time (min)					
		2		5		10	
		$\bar{x}$ ± S_e_	CV (%)	$\bar{x}$ ± S_e_	CV (%)	$\bar{x}$ ± S_e_	CV (%)
Green Tea (n:4)	80	13 ± 2.5	24.3	17 ± 3.6	25.1	17 ± 4.1	28.1
	100	12 ± 3.1	31.0	16 ± 3.8	28.8	17 ± 4.5	32.4
White Tea (n:3)	80	1.8 ± 0.4	22.5	3.3 ± 0.6	19.4	4.3 ± 0.7	17.8
	100	2 ± 1.0	51.1	4 ± 2.0	54.8	4.0 ± 0.7	18.5
Yellow Tea (n:2)	80	4 ± 1.1	21.8	7.6 ± 0.9	10.2	9 ± 5.4	51.9
	100	4.5 ± 0.4	7.5	6.8 ± 0.8	9.6	8 ± 6.3	70.5
Oolong Tea (n:3)	80	2.1 ± 0.5	23.1	1.9 ± 0.5	28.9	2.8 ± 0.4	16.7
	100	2.2 ± 0.3	14.1	2.5 ± 0.7	27.9	4 ± 1.6	41.6
Black Tea (n:6)	80	12 ± 2.8	48.0	21 ± 3.4	27.6	22 ± 2.5	36.5
	100	13 ± 2.5	56.3	21 ± 2.1	27.2	23 ± 4.2	39.8
Pu-erh Tea (n:2)	80	3.1 ± 0.4	10.0	4.2 ± 0.1	1.8	4.6 ± 0.1	2.0
	100	3.2 ± 0.8	22.2	4.0 ± 0.4	9.3	4.6 ± 0.3	4.9

Robust ANOVA. L-theanine levels of tea infusions brewed under the same conditions with different fermentation rates *p* > 0.001. L-theanine levels of tea infusions brewed under different conditions with the same fermentation rates *p* < 0.05. * Owing to the large number of variables, statistical significance levels are not shown in the table and are provided in detail in the explanations of the table.

**Table 5:** Mean ($\bar{x}$), standard deviation (SD), and coefficient of variation (CV) values (%) of the mean L-theanine levels (mg/200 mL) of teas with different serving methods under the same brewing conditions.

Tea Type	Brewing Temperature (°C)	Brewing Time (min)	Tea Bag		Loose-Leaf		Test Statistics *	*p*
			$\bar{x}$ ± ss	CV (%)	$\bar{x}$	CV (%)		
Green Tea (n_TB_:2, n_LT_:2)	80	2	13 ± 4.3	38.1	12 ± 2.7	54.8	0.424	0.713
		5	19 ± 1.1	20.5	15 ± 6.2	52.7	0.946	0.444
		10	20 ± 2.0	22.0	15 ± 5.9	43.8	1.278	0.330
	100	2	12 ± 3.3	37.9	12 ± 5.5	47.9	0.164	0.885
		5	18.1 ± 0.6	24.9	13 ± 5.9	49.9	1.195	0.355
		10	20.5 ± 0.1	33.5	13 ± 4.8	44.7	2.34	0.144
Black Tea (n_TB_:3, n_LT_:3)	80	2	11 ± 1.4	24.7	13 ± 8.5	27.7	−0.475	0.679
		5	20 ± 6.6	14.9	21 ± 5.9	28.0	−0.216	0.840
		10	22 ± 12.0	21.2	22 ± 4.0	27.5	−0.068	0.949
	100	2	13 ± 4.0	26.6	15 ± 11.5	26.7	−0.327	0.760
		5	20 ± 4.3	21.6	23 ± 7.6	26.4	−0.691	0.528
		10	25 ± 13.0	25.3	22 ± 7.0	29.6	0.325	0.761

* TB, tea bag; LT, loose-leaf.

## Data Availability

The original contributions presented in the study are included in the article/supplementary material, further inquiries can be directed to the corresponding author.

## Supplementary Materials

The following supporting information can be downloaded at: https://www.mdpi.com/article/10.3390/foods14132313/s1.

## Abbreviations

GABA	Gamma aminobutyric acid
GC	Gas chromatography
HPLC	High performance liquid chromatography
HPLC-DAD	HPLC-diode array detector
HPLC-MS	HPLC-mass spectrometer
HPLC-UV	HPLC-ultraviolet
LOD	Limit of detection
LOQ	Limit of quantification
POD	Peroxidase
PPO	Polyphenol peroxidase
PVPP	Polyvinylpolypyrrolidone
RP-HPLC	Revere phase-HPLC
