# Supplementary material for: Determination of L-Theanine and Caffeine Contents in Tea Infusions with Different Fermentation Degrees and Brewing Conditions Using the Chromatographic Method
Source: Foods. 2025 Jun 30;14(13):2313. doi: 10.3390/foods14132313 (PMC12248710; doi:10.3390/foods14132313)

Linear Calibration Curves of Standard Caffeine and L-theanine

Linear Calibration Curve of Standard Caffeine

0.1, 0.5, 1, 2 and 3 grams of the powdered caffeine standard were transferred to ultra distilled water in 1000 mL balloon jugs. Magnetic fish was added to each flask and the mixture was stirred at 150 rpm in a magnetic stirrer. The solutions obtained in this way contain 10, 50, 100, 200 and 300 mg/L caffeine in L, respectively.

From each of these standard solutions containing caffeine, 2 mL of each standard solution was taken with the help of a disposable sterile syringe and filtered with syringe tip filters with a pore size of 0.45 μm and the supernatant/clear portions were transferred to the vial. Each of the 5 standards was read on a UV1000 detector set at 275 nm. Linear regression analysis was performed on the difference in absorbance values against changes in caffeine concentration. A calibration graph is plotted showing concentrations (x-axis) versus absorbance differences (y-axis).

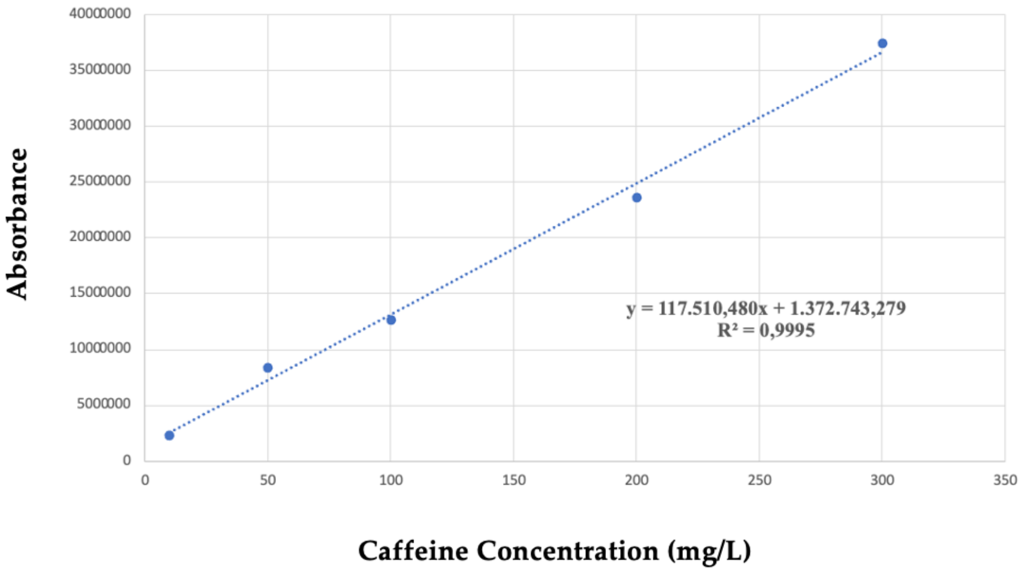

Linear Calibration Curve of Standard L-theanine

0.25, 0.5, 1, 1.5, 1.5, 2 and 2.5 grams of the powdered L-theanine standard were transferred to ultra distilled water in 1000 mL balloon jugs. Magnetic fish was added to each flask and the mixture was stirred at 150 rpm in a magnetic stirrer. The solutions obtained in this way contain 25, 50, 100, 100, 150, 200 and 250 mg/L L-theanine, respectively.

From each of these standard solutions containing L-theanine, 2 mL of each standard solution was taken with the help of a disposable sterile syringe and filtered with syringe tip filters with a pore size of 0.45 µm and the supernatant/clear portions were transferred to the vial. Each of the 6 standards was read on a UV1000 detector set at 210 nm. Linear regression analysis was performed on the difference in absorbance values against changes in caffeine concentration. A calibration graph is plotted showing concentrations (x-axis) versus absorbance differences (y-axis).

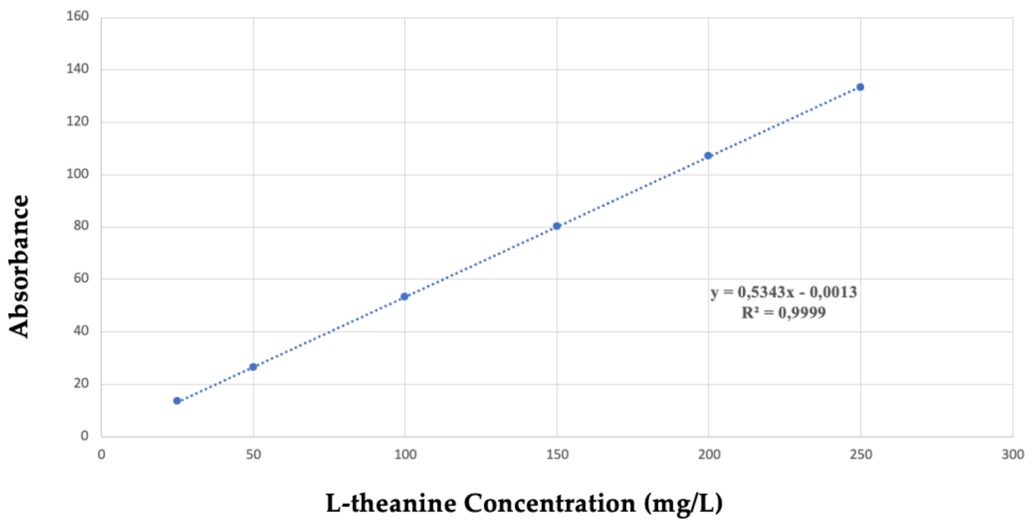

Supplement: Supplementary file 1 [file foods-14-02313-s001.zip › foods-3689502-supplementary.pdf]
